# Naturally Occurring Microbiota Associated with Mosquito Breeding Habitats and Their Effects on Mosquito Larvae

**DOI:** 10.1155/2020/4065315

**Published:** 2020-12-14

**Authors:** H. A. K. Ranasinghe, L. D. Amarasinghe

**Affiliations:** Department of Zoology and Environmental Management, Faculty of Science, University of Kelaniya, Dalugama, Kelaniya, Sri Lanka GQ 11600

## Abstract

Immature mosquitoes are aquatic, and their distribution, abundance, and individual fitness in a particular breeding habitat are known to be dependent on mainly three factors: biotic factors, abiotic factors, and their interaction between each other and with other associated taxa. Mosquito breeding habitats harbor a diversified naturally occurring microbiota assemblage, and the biota have different types of interactions with mosquito larvae in those habitats. Those interactions may include parasitism, pathogenism, predation, and competition which cause the mortality of larvae, natural reduction of larval abundance, or alterations in their growth. Many microbiota species serve as food items for mosquito larvae, and there are also some indigestible or toxic phytoplanktons to larvae. However, when there is coexistence or mutualism of different mosquito species along with associated microbiota, they form a community sharing the habitat requirements. With the available literature, it is evident that the abundance of mosquito larvae is related to the densities of associated microbiota and their composition in that particular breeding habitat. Potential antagonist microbiota which are naturally occurring in mosquito breeding habitats could be used in integrated vector control approaches, and this method rises as an ecofriendly approach in controlling larvae in natural habitats themselves. To date, this aspect has received less attention; only a limited number of species of microbiota inhabiting mosquito breeding habitats have been recorded, and detailed studies on microbiota assemblage in relation to diverse vector mosquito breeding habitats and their association with mosquito larvae are few. Therefore, future studies on this important ecological aspect are encouraged. Such studies may help to identify field characteristic agents that can serve as mosquito controlling candidates in their natural habitats themselves.

## 1. Introduction

Due to the importance of mosquitoes as vectors for diseases in terms of public health, studying their ecological and environmental conditions influencing the abundance of these species is a vital necessity [1]. Determining the larval densities, proliferation, and species assemblage, mosquito habitat ecology plays an important role [2]. Immature mosquitoes are aquatic, and the distribution, abundance, and individual fitness of mosquitoes in a particular breeding habitat are known to be dependent on mainly three factors: biotic factors [3, 4], abiotic factors [5, 6], and their interaction between each other and with other associated taxa [7, 8]. Those interactions included parasitism, pathogenism, predation, and competition. When there is coexistence or mutualism of different mosquito species along with other biotic organisms, they form a community sharing habitat requirements [9].

Larval density in a breeding habitat is affected by different abiotic characteristics of the breeding site such as vegetation, temperature, turbidity, pH, the concentration of ammonia, salinity, nitrite and nitrate, sulfate, phosphate, chloride, calcium, and water hardness [10, 11]. Further, larval densities are controlled by hydrology, light/shade, and nutrient availability also [12]. However, among biotic factors associated with mosquito breeding habitats, several species of bacteria, fungi, unicellular organisms such as protists [13], entomopathogenic nematodes [14], and filamentous fungi [15] are recorded for the infection to mosquito larvae.

There is a diversified naturally occurring microbiota assemblage in mosquito breeding habitats. Microbiota are partly potential food organisms, competitors, and/or potential mosquito predators. However, among them, there may be parasitic or pathogenic microbiota species to mosquito larvae as well. So, some of the microbiota in the habitat act as natural biocontrol agents against mosquito larvae. Microbiota communities associated with mosquito breeding habitats often vary in composition as there are species that are highly sensitive for the changes in nutrient cycling and variable environmental conditions including temperature [16]. Excreta of some animals can be influenced on the structure and functioning of plankton communities as the excreta could act as a significant source of nitrogen and phosphorus. Mosquito larvae were provided with diverse resources to prey on, by the interactions of microinvertebrates [17].

Although vector control strategies have traditionally focused on killing mosquitoes using a variety of synthetic chemical insecticides, the development of insecticide resistance has declined the efficiency of killing mosquitoes. Also, the financial burden of insecticide-based vector control programs is prohibited by the widespread usage of larvicides and adulticides in many countries where mosquito-borne diseases remain endemic [13]. Thus, information on microbiota associated with mosquito breeding habitats and their effects on mosquito larvae is worth investigating with regard to their potential usage in integrated vector control approaches to be used. However, there are only a very limited number of studies and scattered information focused on this aspect; only a limited number of such potential parasitic or pathogenic species have been recorded from those studies.

## 2. Methodology

Reviewing was performed with the use of the six-step methodological approach defined by Arksey and O'Malley [18]. The microbiota association in mosquito breeding habitats and their effects on developing larvae, either as positive or negative influences, were established as the research question to review. Relevant studies were comprehensively searched using a general internet Google search and several electronic databases, including Google Scholar and ResearchGate, meeting abstracts and dissertations. We also searched the Science Citation Index for papers that our initial searches may have missed. A very broad comprehensive search was conducted to gather information as recommended by Arksey and O'Malley [18]. Once the relevant literature was identified, the exclusion and inclusion criteria that were established were applied to the papers.

Inclusion parameters were established; studies on microscopic invertebrate animals such as ciliates, rotifers, and freshwater microcrustaceans and their juvenile stages, mainly comprised of members in the groups of ostracod, copepod, and cladocerans associated with mosquito breeding habitats, were selected. Further, studies on microflora associated with mosquito breeding habitats which mainly included planktonic algae (e.g., green algae, brown algae, and diatoms) and cyanobacteria (blue-green algae) species associated with mosquito breeding habitats were selected. We carefully set parameters for exclusion. Studies related to screening on macrofauna in the mosquito breeding habitats including freshwater fish; macrocrustaceans; larvae of dragonflies/damselflies, mollusks, coleopteran, and hemipteran larvae; and hydrophytes or macrophytes were excluded as they did not fall into the category of the microbiota.

The full text of each article was reviewed to determine its eligibility for our study according to the inclusion and exclusion criteria. After concluding this process, all papers/studies and abstracts which met the inclusion criteria were included into our study. Information was extracted, organized, and sorted according to key themes and issues: pathogenic, parasitic, predatory microbiota, microbiota as competitors and food items, bacteria as microbiota in mosquito breeding habitats, and other microbiota recorded from global studies. Information was managed under those subcategories. Findings were reported using a combination of tables with descriptions according to our themes, in a way that the information clearly links to the extent of the literature and to identify gaps.

## 3. Results

### 3.1. Parasitic and Pathogenic Effects of Microbiota on Mosquito Larvae

Endoparasitic ciliates (Protista: Ciliophora) have been known to infect mosquito larvae since 1921. The first record was by Lamborn, from a sample collected from an earthen pot in Kuala Lumpur reporting the occurrence of *Lambornella stegomyiae* infection in the larvae of *Aedes albopictus* [19].

After about a 74-year gap, the transformation of *Lambornella stegomyiae* trophonts to theronts, the distribution of invasion cysts on larval *Aedes albopictus* cuticle, and the virulence of *L. stegomyiae* to *Ae. albopictus* and *Aedes aegypti* under laboratory conditions were studied by Arshad and Sulaiman [20]. The survival of the parasitic agents (ciliates) is under dry conditions; thus, the encystation of these ciliates is a possible way for the time lap. After excystation, free-swimming stages of these ciliates could be increased easily when the optimum environmental conditions reoccurred. Cysts and the processes of encystation and excystation have been described for many such ciliate species associated with mosquito breeding habitats. Arshad and Sulaiman [20] have found out the transformation of trophonts of the parasitic agent into theronts was induced by a morphogenic agent released from a larval *Ae. albopictus* homogenate. Further, the first transformation was observed 4 hrs after exposure to the larval mosquito homogenate, but most transformations occurred between 12 and 16 hrs. Distribution of invasion cysts on the cuticle of mosquito larvae was not uniform, and most cysts were formed on their abdomen and head. *L. stegomyiae* was highly infective and virulent to *Ae. albopictus* (mortality rate: 99.53%) and *Ae. aegypti* (mortality rate: 90.83%) larvae [20].

After a gap of fifty years of identifying the first parasitic ciliate on mosquito larvae, the second species of *Lambornella* (*L. clarki*) (Ciliophora: Tetrahymenidae) was isolated from newly flooded tree hole-breeding mosquito larvae, *Aedes sirensis* [21]. After twelve years of recording the parasitism of *L. clarki*, Washburn and Mercer [22] have studied the parasitism of newly hatched *Aedes sierrensis* (Diptera: Culicidae) larvae by *L. clarki* following the habitat flooding. As early as 24 hrs after flooding, ciliates initiated the first parasite cycle by forming cuticular cysts on first instar larvae, and by 64 hrs, cysts were observed on larvae. Further, the same study mentioned that among tree-hole populations, the proportion of larvae with *L. clarki* cysts ranged from 2 to 100% at 48 hrs. Ciliates began entering mosquito larval hosts from 48 to 72 hrs after flooding, but some larvae were able to escape from parasitization by molting to the second instar before ciliates penetrate the cuticle [22].

In 1986, Egerter et al. found that the *L. clarki* is dispersed by infected adult mosquitoes. Invasion of the ovaries induces parasitically castrated females to exhibit oviposition behavior and thereby actively disperse ciliates through deposition into water. Adults of both sexes also passively disperse ciliates by dying on water surfaces. However, infected adults were more likely to die on the water than uninfected adults. Ciliates are dispersed by infected adults who can infect larvae and form desiccation-resistant cysts. Parasitism of *L. clarki* indicated a significant biological control potential against container-breeding mosquitoes due to their parasite-induced dispersal by hosts, desiccation-resistant cysts, an active host-seeking infective stage, and high infection and mortality rates [23]. Washburn et al. [24] indicated that induced free-living trophonts of *L. clarki* undergo an asynchronous response in which cells divide and transform into parasitic cells (theronts) that encyst on larval predators. Parasitic ciliates penetrate the cuticle, enter the hemocoel, and ultimately kill their predator-host then. However, Anopheline larvae (*Anopheles barbirostris*, *An. hyrcanus* group, and *An. philippinensis*) breeding in peridomestic ditches was found infected with a *Lambornella* sp. in Northeast India [25].

A ciliate species belonging to another genus, *Tetrahymena pyriformis*, was observed in the body cavity and anal gills of mosquito larvae of bamboo-breeding species *Armigeres dolichocephalus*, *Ar. dentatus*, and *Ar. digitatus* collected from a bamboo forest near Kuala Lumpur by Corliss [26]. The parasitism of *Tetrahymena* to mosquito larvae becomes fatal in heavy infestations which make the larvae transparent, whitish, or opaque. These facultative parasites probably enter via the oral route and invade the hemocoel through the gut wall of mosquito larvae [26]. High concentrations of *Tetrahymena pyriformis* have resulted in high mortalities of *Culex tarsalis* larvae where the concentration is fairly less to achieve the same result for *Aedes aegypti* in the United States [27].

Das [28] has reported that another endoparasitic ciliate, *Chilodonella uncinata*, has been isolated from the infected larval head capsule, antennae, body cavity, anal gills and siphons of Culicine larvae, and Anopheline larvae breed in paddy fields, irrigation channels, marshy areas, wells, ponds, and pools in North India. The natural infestation of *Chilodonella uncinata* has resulted in high mortalities in *Culex tritaeniorhynchus* and *Culex pseudovishnui* larvae collected from paddy fields. *C. uncinata* was found to cause chronic and fatal infection in the natural population of mosquitoes in and around Delhi, North India, while Anopheline larvae were less (14.13%) susceptible to *Chilodonella* infection than Culicine larvae (75.21%). Thousands of motile endoparasitic stage of the ciliate were found packed in body cavities of dead and transparent larvae while numerous cuticular cysts were observed on the cadaver of larvae and pupae [28].

However, a study conducted by Patil et al. [29] revealed inhibition of larval growth, development, and adult emergence of *An. stephensi* larvae due to infection of *Vorticella* sp. The same study further reported that *Vorticella* sp. has the first preference to A*nopheles*, but it could attack other mosquito species like *Aedes aegypti*. Far back in 1950, Micks [30, 31] reported the lethal effect of the ciliate, *V. microstoma*, on *An. quadrimaculatus*. Both the above previous studies suggest that the growth, development, and emergence of mosquito larvae are inhibited by *Vorticella*, resulting in death although the precise reason for that is still unknown. From the microbiota species identified from mosquito breeding habitats in Sri Lanka, *Vorticella microstoma* and *Chilodonella* sp. were found to be effective negatively on *Culex* spp. mosquito larvae and *Zoothamnium* sp. found as an epibiont on *Culex* spp. mosquito larvae. The trophont stages of *V. microstoma* and *Zoothamnium* sp. were found attached to the cuticle of mosquito larvae [32, 33].

### 3.2. Predatory Effects of Microbiota on Mosquito Larvae

The term “predators” of mosquito larvae refers to macro-/microinvertebrates that feed upon mosquito larvae [34, 35]. Naturally occurring Cyclopoids (Subphylum: Crustacea, Sub-class: Copepoda) are able to prey on mosquito larvae [36–40]. The first field trial was carried out with *Mesocyclops aspericornis* against the larvae of *Aedes polynesiensis* Riviere et al. [36] and revealed that they reduce the *Aedes polynesiensis* and/or *Aedes aegypti* by 91-99% in burrows, tree holes, drums, wells, and tires in French Polynesia. But *M. aspericornis* could not effectively reduce the larval population of *Culex* particularly *Cx. roseni* and *Cx. quinquefasciatus*. Thereafter, many field and laboratory trials were conducted with many microbiota species as predators of mosquito larvae, and they are summarized in Table 1.

Determinants of the efficiency of mosquito larval predation are the predator's ability to consume prey from early larvae onwards, high attacking rate, and its preference for target prey instead of other prey types. Additionally, it depends on the predator's preference for advanced instars, as it avoids the compensatory effect of the reduction in competitive interactions among the surviving prey [47, 48].

Besides, Udayanga et al. [46] found that the predatory efficiencies varied significantly among the copepod species; *Mesocyclops leuckarti* showed the highest predatory efficiency for *Ae*. *aegypti* and *Ae*. *albopictus* larvae [46]. *Mesocyclops aspericornis* was the most effective predator of *Aedes* mosquitoes while *Mesocyclops darwini* was less efficient [43]. Rey et al. [44] revealed that cyclopoid copepods were most effective on 1-4-day-old *Aedes* larvae, and further, Chansang et al. [45] found that *Mesocyclops thermocyclopoides* copepods alone were able to produce mortality of 98-100% in the 1st instar larvae of *Ae. aegypti* when the copepod : larvae ratios are ranging from 1 : 1 to 1 : 4.

### 3.3. Microbiota as Competitors of Mosquito Larvae

The competitors also can reduce the survival of mosquitoes by competing for the same food resources. The term “competitors” in relation to mosquitoes refers to invertebrate species who feed upon the same functional food like algae, bacteria, detritus, and protists, as mosquito larvae [49]. These competitors include mainly the species under Subphylum Crustacea, such as cladocerans (Phyllopoda), calanoids (Copepoda), harpacticoids (Copepoda), and ostracods (Ostracoda) [50–52]; and cause a negative impact on mosquito larval populations. Naturally occurring microcrustaceans are potentially effective competitors against mosquito larvae because many species show similar biotope preferences with mosquito larvae [53], and polyphagous activities of mosquito larvae and associated major competitors explain the abundance and coinhabitation of mosquito larvae in breeding habitats [17].

Kroeger et al. [54] highlighted that the larvae of *Cx. pipiens* were found to be spatially associated with competing Cladocera, and they prevent the *Cx. pipiens* colonization. The same study showed Ostracods as abundant microcrustaceans associated with ponds, and their dominance has inhibited the colonization of mosquito larvae in ponds. Nonmosquito competitors such as larvae of Chironomidae and cladocerans were found to limit the abundance of *An. quadrimaculatus* and *Cx. pipiens* to a great extent in temporary ponds of Northwest Pennsylvania in the USA by Chase and Knight [8].

The potential of cladocerans as controphic competitors of the mosquito *Cx. pipiens* was studied [55] and revealed that the oviposition of mosquitoes was fully inhibited under high densities of a cladoceran, *Daphnia magna*, and there were consequently no mosquito larvae. Mosquito larvae in the presence of cladocerans took two more days to emerge than where predators and competitions were absent [56]. *Daphnia magna* did not significantly affect survival to the pupation of *Cx. pipiens*, but competing for food resources, it increased the time for metamorphosis and reduced size at metamorphosis. Further, they caused a small survival reduction (21.9%) in *Culex longiareolata*, while not affecting time to, or size at, pupation [57].

Ostracoda was identified as both predator and food competitor for mosquito larvae, and it shows a strong negative impact on larval development [54, 55, 58, 59]. The effects of ciliate protists and rotifers on lower trophic level microbial food resources, such as bacteria, small flagellates, and organic particles, in the water column, and on *Cx. nigripalpus* larval development and adult production were studied in the recent past [60]. The authors indicated that ciliates and rotifers, singly or in combination, altered other microbial populations in mosquito breeding habitat and thereby inhibited *Cx. nigripalpus* mosquito growth suggesting that instead of serving as food resources, they competed with early instar mosquito larvae for getting food items.

### 3.4. Microbiota as Food Items for Developing Mosquito Larvae

Microbiota that inhabit aquatic habitats serve as food organisms to developing mosquito larvae. Many undergo a similar trend of surviving the ephemeral nature of the microhabitats and eventually, when the conditions become favorable, serve as competitors sometimes or food organisms to mosquito larvae. Depending on the larval species, food items include many microbiota species such as bacteria, fungi, and protists, diatoms, microcrustaceans, cyanobacteria, and unicellular or filamentous algae [61, 62]. Protozoans and rotifers are relatively smaller in their size in which 50-250 *μ*m in length coincides with the waterborne particles ingested by mosquito larvae while filter-feeding [63].

The availability of sufficient food sources determines the proliferation of mosquitoes, affecting them positively most of the time and negatively sometimes. Larval immature survivorship and their developmental rate depend on the quality and quantity of their food. The mosquito adult emergence, body size, response to repellents and insecticides, survival, sexual maturity, fecundity, egg production, and longevity of the adult female and more importantly vector competence are also influenced by the availability of the food resources for larval development [64].

There are at least 200 species of phytoplankton associated with mosquito breeding habitats, and larvae extensively feed upon them [65, 66]. Cyanobacteria have an important role in the diet of mosquito larvae. Kaufman et al. [67] indicated the importance of algal biomass on the growth and development of *An. gambiae* larvae. Although most of the algal species are nutritious food for many species of mosquito larvae, some species are able to kill the larvae if ingested in large quantities. Sometimes, it is possible that they die due to starvation by feeding on indigestible algae. The vector mosquitoes have not developed resistance to these algal toxins.

In particular, Cyanobacteria, the blue-green algae, are able to effect on larval mortality by virtue of toxicity, and some species of green algae (Order Chlorococcales) are able to kill larvae by being indigestible. *Microcystis* sp. showed a significant negative effect on developing mosquito larvae, where the larvae grown in the presence of alga were significantly smaller. Further, species such as *Kirchneriella*, *Scenedesmus*, *Coelastrum*, *Selenastrum*, *Dactylococcus*, and *Tetrallantos* were found virtually indigestible by *Culex*, *Aedes*, and *Anopheles* mosquito larvae, thus reducing their existence and failure to develop successfully in the water where certain species of closely related green algae in the order Chlorococcales are the main source of food [42, 68].

Further, the green alga *Kirchneriella irregularis* could kill *Ae. albopictus* larvae in container breeding habitats in Hawaii due to starvation as they were unable to digest *Kirchneriella*. In order to kill the larvae, there is no need for the *Kirchneriella* to be highly abundant but abundant enough to predominate in larval guts to the exclusion of other food [42]. Marten [69] has reported that many species of *Scenedesmus* were found to kill the larvae. However, in a recent study done in 2017, *Scenedesmus* species were encountered from both larval gut and in larval habitats; its larvicidal property is yet to be confirmed there [70].

Rejmankova et al. [71] found that *An. albimanus* larval densities in cyanobacteria (blue-green algae) mats were relatively high in both wet and dry seasons, concluding that these cyanobacteria mats provide suitable habitats for mosquito larvae. The number of cyanobacterial cells ingested and digested by mosquito larvae was dependent on the cyanobacterial strain and varied with the mosquito species associated [72]. Cyanobacteria species associated with *Anopheles albimanus* larvae from southern Chiapas, Mexico, were studied and revealed the presence of *Phormidium* sp., *Oscillatoria* sp., *Aphanocapsa littoralis*, *P. animalis*, *Lyngbya lutea*, and *Anabaena spiroides*. However, *Aphanocapsa littoralis* were associated with habitats of relatively lower larval abundance, and higher cyanobacteria abundance was observed from estuaries, irrigation canals, river margins, and mangrove lagoons [73].

From a study carried out in Finland, Cyanobacterium *Oscillatoria agardhii* and *Anabaena circinalis* were found as highly toxic to *Aedes aegypti* larvae [74]. Further, the toxin was found to be water-soluble, and fourth and second instar larvae of *A. aegypti* showed 24 h LC_50_ values as 8.7 and 6.1 *μ*g live cells/mL, respectively. However, larval production could be reduced in the absence of some algal species as well. Bond et al. [75] at Chiapas, Mexico, reported that *An. pseudopunctipennis* breeding was reduced by removing a filamentous chlorophyte green alga, *Spirogyra*, from their breeding sites. The extraction of this alga brought about a striking decline in the density of *An. pseudopunctipennis* larvae sustained for about six weeks and thus a concurrent reduction in the adult population.

Cyanobacteria, numerous unicellular and filamentous algae, zooflagellates, and other protozoans, rotifers, crustaceans, organic debris, unspecified inorganic materials, spores, and insect scales were identified by the dissected guts of several mosquito species belonging to five genera [76]. Dissections of the larvae of *A. punctipennis*, *A. quadrimaculatus*, and *A. crucians* showed that all three species were indiscriminate feeders and that none has a characteristic plankton food in their guts, and the places in which *A. punctipennis* breeds throughout the season were identified as always deficient in plankton in which alimentary tracts of some contained only particles of clay and silica [77]. However, Kaufman et al. [67] indicated the importance of algal biomass to the growth and development of *Anopheles gambiae* larvae.

### 3.5. Bacteria as Microbiota Associated with Mosquito Breeding Habitats

The bacteria in mosquito breeding waters can affect ovipositing mosquitoes, have effects on larval development, and can modify larval and adult mosquito gut bacterial composition [78]. Mosquitoes are exposed to a variety of bacterial species in their habitats. Bacteria inhabiting in larval habitats have been considered as the most important that comprise the food of mosquito larvae by previous studies carried out [61, 79, 80]. Bacteria act as the most abundant microbiota present in mosquito larval diets and sometimes can even be the major nutritional source for their growth and development. Mosquito larval growth is possible in cultures of bacteria alone [62].

Rozeboom [81] found that *Aedes aegypti* larvae could not develop in bacteria-filtered water, revealing that bacteria are indispensable for the mosquito larval development.

Further, higher larval mortalities were observed in water treated with antibiotics [82]. Besides, many bacteria have been shown to either attract [83, 84] or repel (Juan [85]) gravid mosquitoes to potential breeding sites. Mosquitoes preferred to oviposit on unmodified substrates from natural larval habitats containing live microorganisms and microbial populations in breeding sites. They were found to produce volatiles, specific bacteria-associated carboxylic acids and methyl esters that serve as potent oviposition stimulants for gravid *Ae. aegypti* [84]. However, oviposition was significantly reduced when the bacterial colonies of *Stenotrophomonas maltophilia* was present and oviposition was neither reduced nor enhanced with the presence of bacterial species, *Pseudomonas putida* or *Pseudomonas alcaligenes* (Juan [85]).

Bacterial species that are present in mosquito larval habitats are acquired from the aquatic larval stage, and they are established in the midgut of mosquito larvae, exhibiting different functional tasks and retaining in the gut as symbiotic species. Many recent studies have used culture-dependent and culture-independent approaches to characterize the microbial communities in different mosquito species including *Aedes*, *Culex*, *Anopheles*, and *Mansonia* mosquitoes. The members of Enterobacteriaceae (e.g., Enterobacter), Erwiniaceae (e.g., Pantoea), and Bacillaceae (e.g., *Bacillus*) have been identified as the most frequently described bacteria from the gut of adult *Aedes* spp. [86–90]. Several studies were conducted on symbiotic bacteria in *Anopheles* mosquitoes and species belonging to several genera; *Pseudomonas*, *Alcaligenes*, *Bordetella*, *Myroides*, *Aeromonas*, *Acinetobacter*, *Bacillus*, *Chryseobacterium*, *Delftia*, *Exiguobacterium*, *Kurthia*, *Microbacterium*, *Staphylococcus*, *Thorsellia*, and *Variovorax* have been identified [91]. Besides, few studies about the bacteria species in vector mosquitoes have been conducted [92–95]. Extensive dispersal and evolutionary success of mosquitoes are widely motivated by these symbiotic relationships with microbes and mosquito larval stages. Adult mosquitoes have been shown to contain gut bacteria found in their breeding waters [96, 97].

These microbial communities and their roles in mosquito biology have been more broadly studied, and midgut microbiota of mosquitoes were proven to play various important roles in immunity, food digestion, fertility, and fecundity, thereby affecting larval growth, adult fitness, vector populations, and disease prevalence [98]. In addition, studies have summarized the positive and negative effects of these gut microbial communities on vector competency through interaction with hosts and parasites [88, 99]. The resident bacteria were shown to promote or assist the gut infection of incoming pathogens of mosquitoes or augment the immune responses of the mosquito [93, 100–107] or impair pathogen infection through competition for resources [108].

Bacteria species associated with waters in a variety of mosquito breeding habitats have been investigated from previous studies [97, 109–112]. Characterization of bacterial communities in breeding waters of *Anopheles darlingi* in Manaus revealed that *An*. *darlingi* can develop in breeding waters with different surface-water bacteria but that the common microbiota found in all breeding sites might indicate or contribute to a suitable habitat. *Escherichia*/*Shigella*, *Staphylococcus*, and *Pseudomonas* and all sites were found, and bacteria species composition was dominated by the bacteria species that belonged to phylum Proteobacteria and Firmicutes [78]. In Thailand and Laos, a higher abundance of *Escherichia coli* in breeding waters was strongly correlated to the presence of *Ae. aegypti* mosquitoes [113].

### 3.6. Other Records on Microbiota Associated with Mosquito Breeding Habitats

Many other microbiota species/taxa were recorded from a variety of breeding habitats globally. They have been summarized in Table 2.

## 4. Conclusion

Potential biocontrol of mosquito larvae with naturally occurring microbiota associated as predators/pathogens and other biocontrol agents would be a more effective and ecofriendly approach. Therefore, with the available literature, it is evident that the abundance of mosquito larvae is inversely related to the densities of associated microbiota that are potential antagonists. Mosquito indigestible or toxic phytoplanktons could serve as a field characteristic agent against mosquito larval control. To date, only a small number of species of the microbiota that inhabit in mosquito breeding habitats have been recorded, and detailed studies on microbiota assemblage in relation to diverse vector mosquito breeding habitats and their association with mosquito larvae are few. Therefore, future studies on this ecological aspect are encouraged. Such studies may help health researchers, entomologists, policy makers, and practitioners for developing strategies for the management of vector mosquito larvae.

## Figures and Tables

**Table 1:** Recorded predatory cyclopoid species associated with mosquito breeding habitats.

Microbiota species	Mosquito species tested against	References
*Mesocyclops aspericornis*	*Aedes aegypti* and *Aedes polynesiensis*	Lardeux et al. [36]
*Mesocyclops* spp.	*Anopheles albimanus*	Marten [41]
*Mesocyclops longisetus* and *Macrocyclops albidus*	*Anopheles* spp. and *Culex quinquefasciatus*	Marten et al. [37]
*Mesocyclops longisetus*	*Aedes aegypti*	Marten et al. [37]
*Mesocyclops longisetus* and *Mesocyclops albidus*	*Anopheles* spp. and *Culex quinquefasciatus*	Marten et al. [37]
*Mesocyclops longisetus*	*Aedes aegypti*	Marten et al. [37]
*Mesocyclops leuckarti pilosa*	*Aedes albopictus*	Marten [42]
*Mesocyclops aspericornis*, *Mesocyclops australiensis*, *Mesocyclops darwini*, and *Mesocyclops notius*	*Aedes* spp.	Brown et al. [43]
*Macrocyclops albidus*	*Aedes* spp.	Rey et al. [44]
*Mesocyclops thermocyclopoides*	*Ae. aegypti*	Chansang et al. [45]
*Mesocyclops leuckarti* and *Mesocyclops scrassus*	*Aedes aegypti* and *Aedes albopictus*	Udayanga et al. [46]

**Table 2:** Other global records on microbiota associated with mosquito breeding habitats.

Description on study	Identified microbiota species and effects on mosquito larvae	References
Microinvertebrates coinhabited with mosquito larvae of *Ae. vittatus*, *An. gambiae*, *Cx. macfiei*, *Cx. perfidiosus*, *Cx. pipiens*, and *Cx. simpsoni* were identified from patchy rock pools on inselbergs within Kaduna state, Nigeria	(i) Protists: *Paramecium caudatum*, *Pleurotricha* sp., and *Chlamydomonas reinhardtii* (ii) Rotifers: *Brachionus plicatilis* and *Philodina* sp. (iii) Microcrustaceans: *Ephemeroporous barroisi*, *Bosmina longirostris*, *Daphnia pulex*, *Diaphanosoma birgei*, *Diaphanosoma brachyurum*, *Laptonopsis occidentalis*, *Macrothrix pulex*, *Macrothrix rosea*, *Moinodaphnia macleayi*, *Moina macrocopa*, *Sida crystallina*, *Bradleystrandesia reticulata*, *Candona intermedia*, *Candona parallela*, *Cypria obesa*, *Heterocypris incongruens*, *Potamocypris hyboforma*, *Cyclops* sp., and *Macrocyclops* sp.	Obi et al. [17]
The associated invertebrate taxa with mosquito larvae were studied in temporary ponds of wetland areas in Germany	(i) Ciliates, rotifers, microcrustaceans (Cladocera, Copepoda, and Ostracoda), isopods (*Asellus*) (ii) Microcrustaceans were identified as the most abundant and most frequently encountered invertebrates (iii) The abundance of *Aedes* spp. was affected by the presence of *Ceriodaphnia* spp., *Chydorus* spp., *Daphnia* spp., *Simocephalus* spp., Calanoida, and larvae of Chironomidae as they competed efficiently with mosquito larvae for food resources (iv) Cyclopoida act as antagonists while Zygoptera and Dytiscidae which were known as strict predators exerted the smallest influence	Elono et al. [114]
Prevalence of microfauna associated with different mosquito breeding habitats in Mawanella area in Sri Lanka	(i) *Coleps hirtus*, *Zoothamnium* sp., *Vorticella* sp., *Chaetonotus* sp., *Ichthydium* sp., *Lecane* sp., and *Rotaria* sp.	Amarasinghe and Rathnayaka [115]
Ecological characterization of *Ae. aegypti* larval habitats in artificial water containers in Girardot, Colombia	(i) Three main taxa of algae were found, Bacillariophyceae, Chlorophyceae, and Cyanobacteria. The diversity of Bacillariophyceae was higher in the larval habitats, and only Cyanobacteria were positively related to the abundance of immature stage of *Ae. aegypti* (ii) *Oscillatoria*, *Dactylococcopsis*, *Nostoc*, *Synedra*, *Scenedesmus*, *Pinnularia*, *Cymbella*, *Meridium*, *Navicula*, and *Dictyosphaerium* were identified as the most abundant algal genera (iii) *Oscillatoria*, which belonged to Cyanobacteria, had the greatest abundance (iv) The only zooplankton found were some rotifers, in very small numbers, and in only a few samples	Garcia-Sánchez et al. [70]
Biotic factors associated with the presence of *Anopheles arabiensis immatures* and their abundance in naturally occurring and manmade aquatic habitats at low altitudes in remote areas in Reunion Island	(i) Main variables associated with the presence of *An. arabiensis* larvae in habitats were green algae and the *Cyperaceae* plant family (ii) *An. arabiensis* larvae were associated with approximately 13 species of macroinvertebrates	Gouagna et al. [116]
Microbiota associated with irrigated rice fields in Sri Lanka	(i) Identified 94 species of invertebrates belonged to 10 phyla	Bambaradeniya et al. [117]
Microbiota associated with a variety of mosquito breeding habitats in Kurunegala and Gampaha districts, Sri Lanka	(i) Forty-five microbiota species/taxa from Gampaha district and 44 microbiota species/taxa from Kurunegala district were identified which belonged to Amoebozoa, Arthropoda, Bacillariophyta, Ciliophora, Charophyta, Chlorophyta, Sarcodina, Cyanobacteria/Cyanophyta, Euglenozoa, Ochrophyta/Heterokontophyta, and Rotifera	Ranasinghe and Amarasinghe [33] and Amarasinghe and Ranasinghe [32]
Microbiota associated with mosquito larvae collected from different larval habitats in Mysore	(i) Had a greater proposition of species belonged to the phylum Bacillariophyceae compared to other species in the phyla of Chlorophyceae, Cyanophyceae, Desmidaceae, and Euglenophyceae (ii) *An. stephensi* mosquito larvae were associated with the filamentous chlorophyte green algae, *Spirogyra* spp., which was served as food for them (iii) Filamentous cyanobacterium, *Oscillatoria* species, was encountered in breeding sources of *Cx. quinquefasciatus*	Charles et al. [118]
Algae species associated with mosquito breeding habitats in Michigan state	(i) Anopheline larvae were strongly associated with naturally occurring algae in their habitats	Wallace and Merritt [119]
